# Forecasting Ocean Chlorophyll in the Equatorial Pacific

**DOI:** 10.3389/fmars.2017.00236

**Published:** 2017-07-26

**Authors:** Cecile S. Rousseaux, Watson W. Gregg

**Affiliations:** 1 Global Modeling and Assimilation Office, NASA Goddard Space Flight Center, Greenbelt, MD, United States; 2 Universities Space Research Association, Columbia, MD, United States

**Keywords:** enso, chlorophyll, phytoplankton, forecast, biogeochemical modeling

## Abstract

Using a global ocean biogeochemical model combined with a forecast of physical oceanic and atmospheric variables from the NASA Global Modeling and Assimilation Office, we assess the skill of a chlorophyll concentrations forecast in the Equatorial Pacific for the period 2012–2015 with a focus on the forecast of the onset of the 2015 El Niño event. Using a series of retrospective 9-month hindcasts, we assess the uncertainties of the forecasted chlorophyll by comparing the monthly total chlorophyll concentration from the forecast with the corresponding monthly ocean chlorophyll data from the Suomi-National Polar-orbiting Partnership Visible Infrared Imaging Radiometer Suite (S-NPP VIIRS) satellite. The forecast was able to reproduce the phasing of the variability in chlorophyll concentration in the Equatorial Pacific, including the beginning of the 2015–2016 El Niño. The anomaly correlation coefficient (ACC) was significant (*p* < 0.05) for forecast at 1-month (*R* = 0.33), 8-month (*R* = 0.42) and 9-month (*R* = 0.41) lead times. The root mean square error (RMSE) increased from 0.0399 μg chl L^−1^ for the 1-month lead forecast to a maximum of 0.0472 μg chl L^−1^ for the 9-month lead forecast indicating that the forecast of the amplitude of chlorophyll concentration variability was getting worse. Forecasts with a 3-month lead time were on average the closest to the S-NPP VIIRS data (23% or 0.033 μg chl L^−1^) while the forecast with a 9-month lead time were the furthest (31% or 0.042 μg chl L^−1^). These results indicate the potential for forecasting chlorophyll concentration in this region but also highlights various deficiencies and suggestions for improvements to the current biogeochemical forecasting system. This system provides an initial basis for future applications including the effects of El Niño events on fisheries and other ocean resources given improvements identified in the analysis of these results.

## INTRODUCTION

Forecast models of atmospheric conditions have considerably improved over the past few decades and are routinely used to predict weather patterns including hurricanes, winds and other potentially threatening conditions. Natural processes in the atmosphere, ocean and land can each influence climate in sometimes predictable ways. Developing forecasting systems for ocean biogeochemical processes is a scientific challenge that has important implications in the management of marine ecosystems and resources. One of the challenges of improving subseasonal to seasonal forecasting skill is to identify and characterize sources of subseasonal to seasonal natural modes of variability (e.g., El Niño Southern Oscillation), slowly varying processes (e.g. ocean biogeochemistry), and external forcing (e.g., winds, radiation).

Most oceanographic forecasts emphasize physical conditions (e.g., temperature, mixing), ocean biogeochemical forecasts are less common and have mostly focused on the prediction of algal blooms and hypoxia (e.g., Wynne et al., 2005; Greene et al., 2009; Stumpf et al., 2009; Evans and Scavia, 2010). Various approaches have been developed to predict biogeochemical variables from statistical relationships with temperature, wind speed and other variables to the use of more complex numerical models. A typical application of these biogeochemical forecasts is the prediction of Harmful Algal Blooms (e.g., Stumpf et al., 2009; Raine et al., 2010). One example is the Eastern Gulf of Mexico Harmful Algal Bloom Operational Forecast System (GOMX HAB-OFS) developed by NOAA to follow the development of a toxic dinoflagellate, *Karenia brevis*, that produces Neurotoxic Shellfish Poisoning, kills fishes and marine mammals and leads to health and economical losses resulting from respiratory irritation in the waters off Florida. This forecasting system relies on satellite ocean color and transport direction data from satellite imagery combined with in situ samples. They issue semi-weekly bulletins that serve as decision support tools for coastal resource managers, federal and state agencies, public officials, and academic institutions (Kavanaugh et al., 2016). The forecast was expanded to other regions and the system is described in several papers (e.g., Stumpf et al., 2003, 2009; Tomlinson et al., 2004). Other examples of biogeochemical forecast efforts include the forecast of hypoxia zone in the Gulf of Mexico (Scavia et al., 2003), net primary production in the tropical Pacific (Séférian et al., 2014), annual salmon yields (Scheuerell and Williams, 2005), sardines distribution (Kaplan et al., 2016), seasonal distributions of southern Bluefin tuna (Hobday et al., 2011; Eveson et al., 2015) and coral bleaching (Goreau and Hayes, 2005).

While some of these forecasting systems rely on satellite ocean color data, others rely on biochemical variables that cannot be directly derived from ocean color data or that do not have statistical relationship with variables that can be derived from satellite data (e.g., nutrient, oxygen concentration). Furthermore, satellite data can have large gaps (e.g., clouds, aerosols, interorbital gaps, high solar zenith angles) that do not allow for a systematic and complete coverage of the area of interest. Here we combine an established biogeochemical model with a seasonal forecast of atmospheric and ocean conditions to provide a 9-month forecast of total chlorophyll in the Equatorial Pacific for the period 2012–2015. The assimilation of satellite ocean color to provide the initial conditions for the forecast ensures the best use of the data available, while the forecast provides a complete coverage of the chlorophyll concentration (among other variables) for a 9-month forecast. The skill of the forecasting system is assessed by comparing the total chlorophyll to those from the satellite Suomi-National Polar-orbiting Partnership Visible Infrared Imaging Radiometer Suite (S-NPP VIIRS).

## MATERIALS AND METHODS

The NASA Ocean Biogeochemical Model (NOBM) is a three dimensional biogeochemical model of the global ocean coupled with a circulation and radiative model (Gregg et al., 2003; Gregg and Casey, 2007). NOBM has a near-global domain that spans from −84° to 72° latitude at a 1.25° resolution in water deeper than 200 m. NOBM is coupled with the Poseidon ocean general circulation model. The Poseidon model (Schopf and Loughe, 1995) is a reduced gravity ocean model with 14 layers in quasi-isopycnal coordinates forced by wind stress, sea surface temperature, and shortwave radiation (Gregg and Casey, 2007). The NOBM contains 4 explicit phytoplankton taxonomic groups (diatoms, cyanobacteria, chlorophytes and coccolithophores), 3 detritus components (silicate, nitrate/carbon and iron), 4 nutrients (nitrate, silicate, iron and ammonium) and one zooplankton group. The growth of phytoplankton is dependent on total irradiance, nitrogen (nitrate + ammonium), silicate (for diatoms only), iron and temperature (see Rousseaux and Gregg, 2015 for more details). Surface photosynthetically available radiation is derived from the Ocean-Atmosphere Spectral Irradiance Model (OASIM; Gregg and Casey, 2009).

A spin-up run of 100 years has been shown to produce stable initial conditions for biological variables (Gregg and Rousseaux, 2014). The NOBM model is then run for 14 years using ocean and atmospheric variables as forcing from the Modern-Era Retrospective analysis for Research and Applications (MERRA, Rienecker et al., 2011) and ocean chlorophyll data from Sea-Viewing Wide Field-of-View Sensor (SeaWiFS) and Moderate-resolution imaging spectroradiometer (MODIS)-Aqua in data assimilation mode (Gregg and Rousseaux, 2014). Starting in 2012, the model assimilates chlorophyll data from S-NPP VIIRS and uses transient MERRA data to force the circulation model. The assimilation of satellite chlorophyll uses a multivariate methodology where the nutrients are adjusted corresponding to the chlorophyll assimilation using nutrient-to-chlorophyll ratios embedded in the model (Rousseaux and Gregg, 2012). The difference between the chlorophyll assimilation results and the prior chlorophyll produced by the model (the analysis increments) are used to adjust the nutrient concentrations. The multivariate assimilation is applied to silica and dissolved iron, as well as nitrate. These conditions are used as initial conditions for each forecast (using the month prior to the start of the forecast). The forcing data used for the forecast include zonal and meridional wind stress, sea surface temperature and shortwave radiation. These forecast files are produced by the NASA Global Modeling and Assimilation Office (GMAO) using the GEOS-5 system (https://gmao.gsfc.nasa.gov/weather_prediction/). These forecasted atmospheric and ocean variables are currently provided to the North American Multi-Model Ensemble (NMME) prediction project, as well as to other national (International Research Institute for Climate and Society, IRI) and international (Asia-Pacific Climate Center, APCC) ensemble seasonal forecasting efforts (Borovikov et al., in review).

**FIGURE 1:**
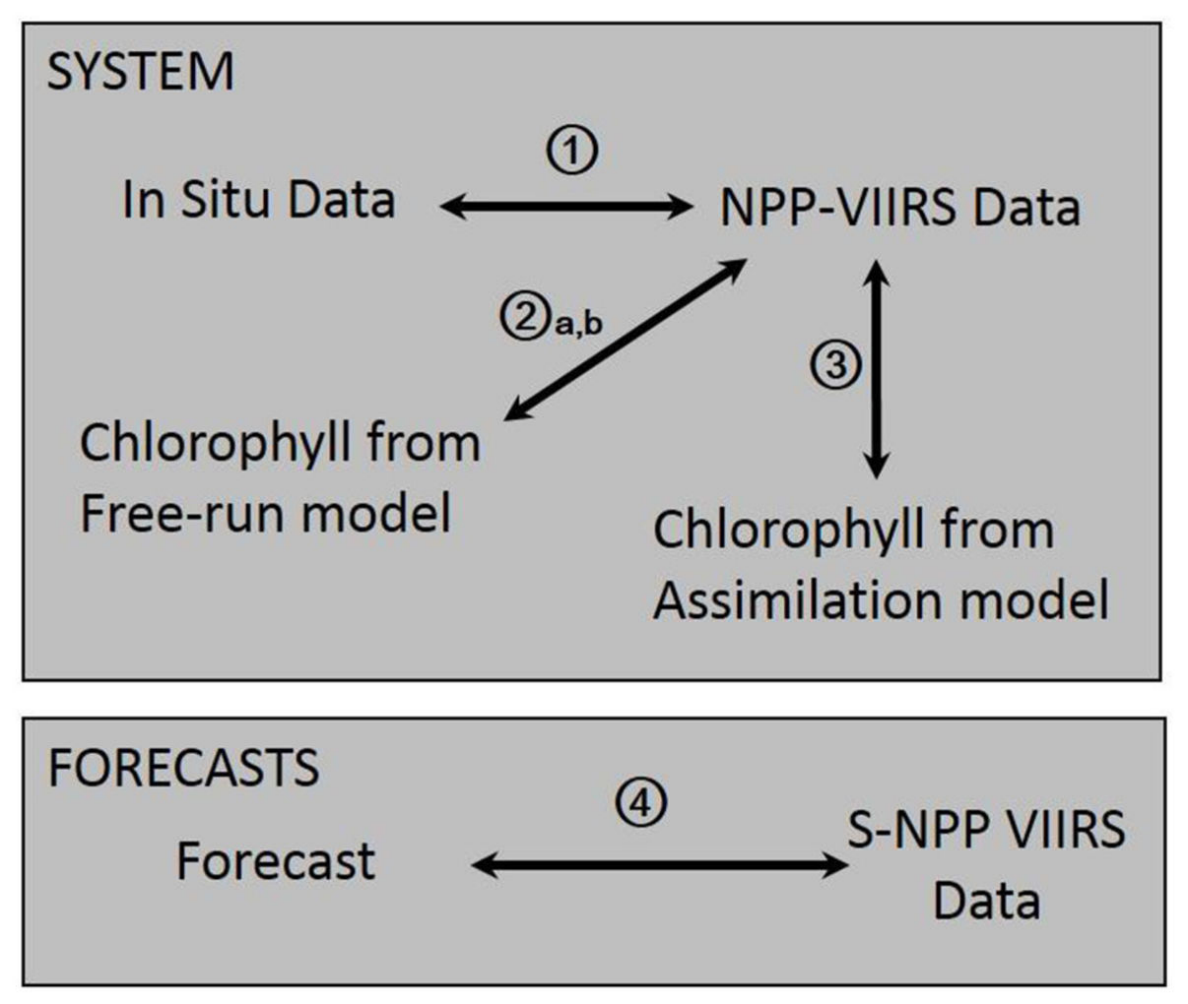
Diagram describing the different procedures used to characterize bias and uncertainties in the system and forecasts described in this study.

The bias and uncertainties in the system are assessed by (1) comparing the satellite ocean chlorophyll used for validation and data assimilation to in situ data, (2) comparing the chlorophyll concentration from a free-run model (without data assimilation) to satellite ocean color and (3) comparing the chlorophyll concentration from a run assimilating satellite chlorophyll with those from the satellite (Figure 1). The in situ data used to evaluate the bias and uncertainties in the S-NPP VIIRS chlorophyll include data collected from the National Oceanographic Data Center (Gregg and Conkright, 2002), NASA in situ database (Werdell and Bailey, 2002; Werdell et al., 2003), and Atlantic Meridional transect (Aiken et al., 2000) archives (Gregg et al., 2009). The quality of the biogeochemical system used is then assessed using a hindcast from 2012 to 2015 forced using MERRA data (procedure 2a, b on Figure 1). The uncertainties in this system are evaluated by comparing the chlorophyll concentration in the Equatorial Pacific from this run with those from S-NPP VIIRS. To evaluate the effects of the forcing data on the chlorophyll concentration estimates, we then compare a free-run model forced by transient MERRA forcing data with one forced by climatological MERRA data. Finally we compare the monthly chlorophyll concentration from the assimilation run to the monthly concentration from S-NPP VIIRS (procedure 3 on Figure 1). Bias is quantified by averaging the monthly percent difference between the chlorophyll concentration from the model (free-run and assimilating run) and the satellite chlorophyll concentration for the period 2012–2015 and the standard error is calculated. The uncertainty is quantified using a correlation coefficient. A statistically significant correlation coefficient is defined as one with a *p*-value smaller than 0.05.

**FIGURE 2:**
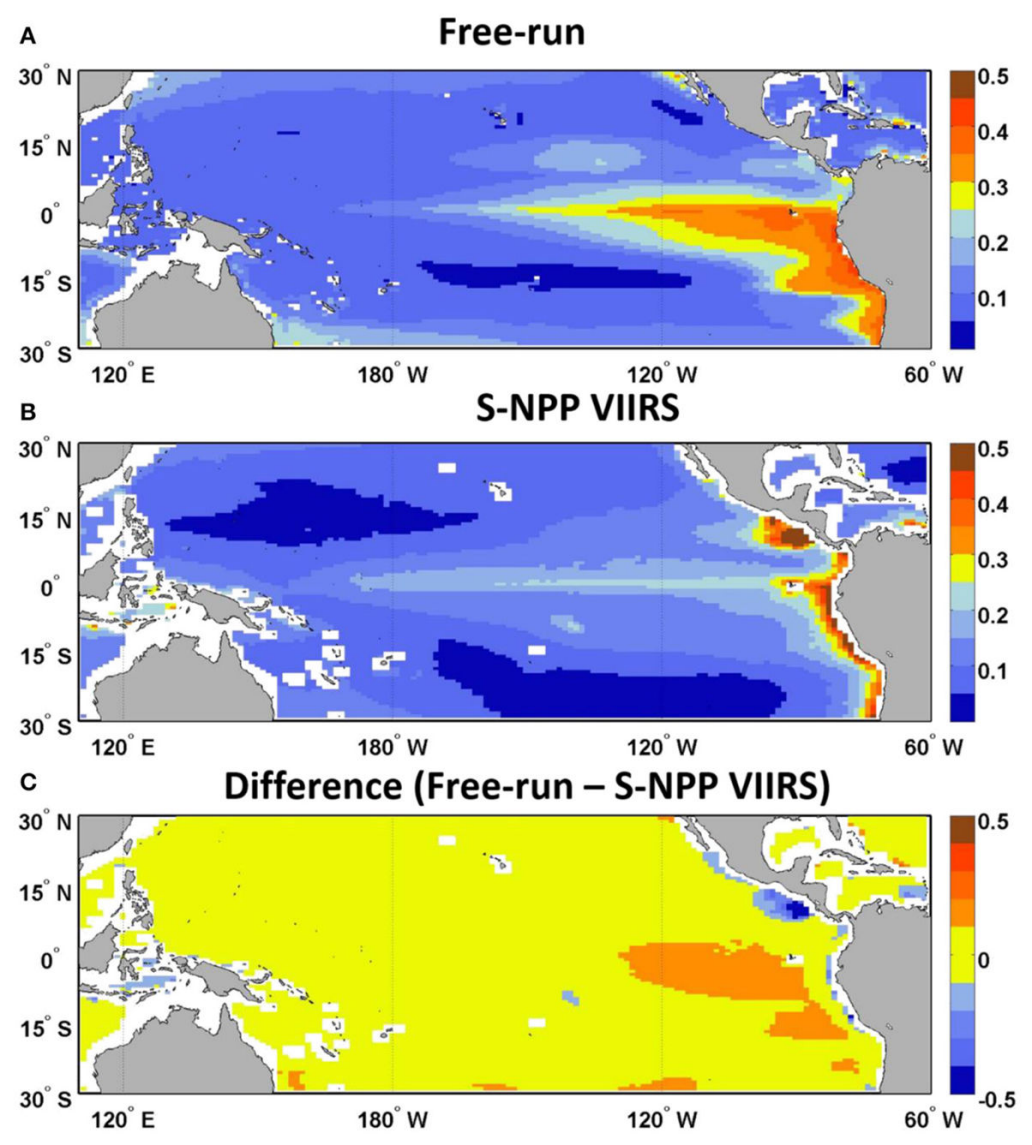
Climatology of chlorophyll concentration (μg chl L^−1^, 2012–2015) map of (**A**) the free-run model, (**B**) S-NPP VIIRS, and (**C**) the difference between the free-run model and S-NPP VIIRS in the Equatorial Pacific.

The skill of the various forecasts is assessed using three metrics: (1) the percent difference between the NPP-VIIRS chlorophyll data and the forecast (bias) (procedure 4 on Figure 1), (2) the anomaly correlation coefficient (ACC) and (3) the root mean square error (RMSE). The anomaly correlation coefficient provides information on the linear association between forecast and observations but is insensitive to biases and error in variances. It is calculated as between the model prediction (p) and satellite observation (o) of chlorophyll over N months (*N* = 38) and computed as:

$$\mathrm{ACC}=\frac{\sum \left(p-\bar{p})(o-\bar{o}\right)}{\sqrt{\sum {\left(p-\bar{p}\right)}^{2}\sum {\left(o-\bar{o}\right)}^{2}}}$$

The RMSE measures the magnitude of the error, is sensitive to large values but does not indicate the direction of the error. It is calculated as:

$$\mathrm{RMSE}=\sqrt{\frac{1}{N}\sum {\left[(p-\bar{p})(o-\bar{o})\right]}^{2}}$$

where *p̄* and *ō* are the temporal averages of chlorophyll.

A total of 38 retrospective forecasts were run, each for a 9-month period. The first forecast started in March 2012 and the last forecast started in April 2015. The percent difference between the satellite and the forecast chlorophyll quantifies the mean error in the forecast. It allows us to assess whether the forecast has on average a positive or a negative bias.

## RESULTS AND DISCUSSION

### Assessing the Skill of the Model System

The first source of uncertainty reflects the inherent bias of satellite-derived chlorophyll concentration and is assessed by comparing the S-NPP VIIRS chlorophyll to in situ fluorometric chlorophyll data. For the period from 2012 to 2014, the global chlorophyll from S-NPP VIIRS compared favorably to in situ chlorophyll (bias = 11.8%, semi-interquartile range = 27.9% and *R* = 0.86; Table 1).

The second source of uncertainty lies in how well the model simulates chlorophyll concentration. This source of uncertainty is assessed by comparing the chlorophyll concentration (Toggweiler et al., 1991) from the free-run model (no data assimilation but uses transient forcing conditions from MERRA) with the corresponding satellite ocean color data. For the period from 2012 until 2015, monthly chlorophyll concentration from the free-run model were significantly correlated to those from the satellite ocean color (S-NPP VIIRS, *R* = 0.72, *p* < 0.05; Table 1). The chlorophyll from the free-run model was on average within 27.87 ± 1.72% (average ± standard error) of the S-NPP VIIRS chlorophyll. Chlorophyll fields in the Equatorial Pacific showed agreement with satellite data (Figure 2). The model reproduces the main features observed by the satellite ocean color. The consistent positive bias in chlorophyll concentration in the Equatorial Pacific in the free-run model suggest that the upwelling in the Equatorial Pacific in the model is overestimated and therefore leads to higher chlorophyll concentration than those observed. The overprediction of the upwelling in the Equatorial Pacific in models has been suggested for some time (e.g., Toggweiler et al., 1991; Zheng et al., 2012). In some other areas, such as along the South America coastline as well as in the region of the Costa Rica Dome, the chlorophyll concentration from the free-run model was underestimated. This is most likely due to the nature of the reduced gravity circulation model. The model therefore does not include topographic effects, nor does it allow the representation of cross-shelf advection and convection.

In the Equatorial Pacific, the monthly chlorophyll concentration from a run assimilating S-NPP VIIRS chlorophyll data was significantly correlated (*R* = 0.95, *P* <0.01; Table 1) and on average within 12.34 ± 0.52% of the S-NPP VIIRS chlorophyll concentration. The assimilation of satellite chlorophyll to provide the initial conditions used for the forecast is therefore an improvement over using the initial conditions provided by the free-run model without data assimilation. We therefore use this set-up to provide the initial conditions for the forecasting systems.

**FIGURE 3:**
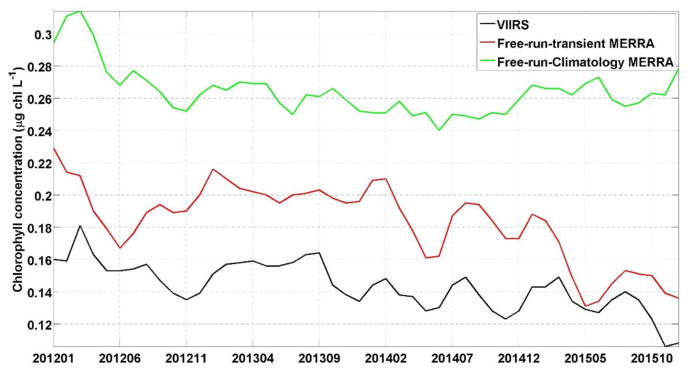
Time series of chlorophyll concentration (μg chl L^−1^) for NPP-VIIRS (black), free-run model with transient MERRA forcing data (red) and free-run model with a climatological MERRA forcing data (green).

Finally the data used to force the model have their own inherent bias and uncertainties. While this is beyond the scope of this paper, we note that the bias in the forcing data used here have been assessed in other papers (e.g., Rienecker et al., 2011). By comparing the chlorophyll concentration from the free-run model using climatological MERRA forcing data compared to using transient MERRA data we can assess the improvements that such transient forcing data can provide to the system. The chlorophyll concentration from the free-run model using transient MERRA forcing data were considerably closer to the chlorophyll concentration from the S-NPP VIIRS (27.87 ± 1.72%) than the free-run model using climatological MERRA data (85.67 ± 2.77%, Figure 3). This indicates the advantage of using transient forcing data to further improve the initial conditions used for the forecasting system.

### General Skill of the Forecasts

**FIGURE 4:**
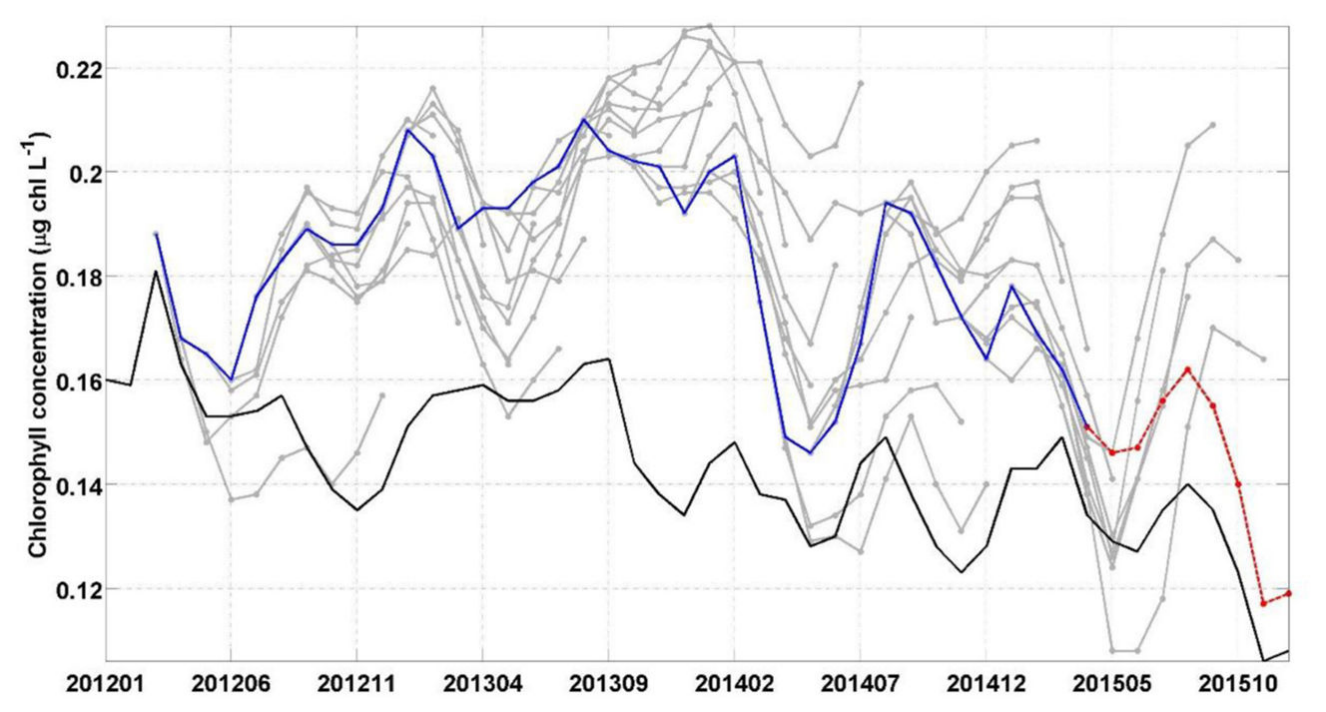
Chlorophyll concentration in the Equatorial Pacific (10°S–10°N) for the period 2012–2015 from S-NPP VIIRS (black), individual forecasts (gray) and the 1-month lead chlorophyll concentration of every forecast (blue). The last forecast is highlighted in red.

**FIGURE 5:**
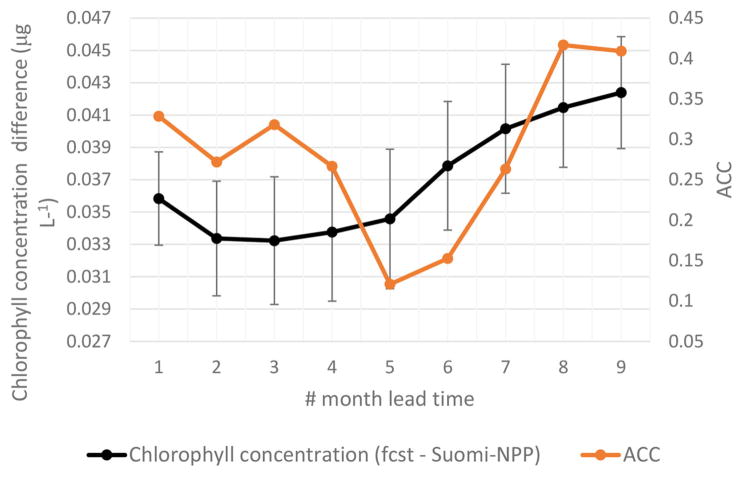
Average difference between forecasted chlorophyll and chlorophyll from S-NPP VIIRS for corresponding month (left axis) and Anomaly Correlation Coefficient (ACC; right axis).

We assess the skill of our forecast by comparing each 9-month forecast to the observed chlorophyll concentration in the Equatorial Pacific from S-NPP VIIRS for the corresponding month. There was a consistent positive bias in the chlorophyll forecasted, as in the hindcast from the free-run model compared with S-NPP VIIRS (Figure 2). Of the 38 forecasts, the average percent difference between the forecasted chlorophyll and the S-NPP VIIRS chlorophyll varied between 23% (3 months lead time, the equivalent of 0.033 μg chl L^−1^) and 30.7% (9 months lead time, the equivalent of 0.042 μg chl L^−1^, Figures 4, 5). Except for the monthly chlorophyll concentration at 5 and 6-month lead time, the chlorophyll concentration from the forecasts were always significantly correlated to those from S-NPP VIIRS (data not shown). The highest correlation coefficient was observed at 8-month lead time (*R* = 0.53, *p* < 0.01).

**FIGURE 6:**
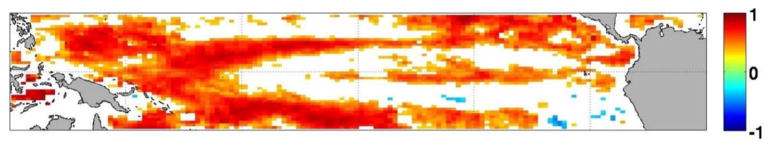
Anomaly correlation coefficient between the forecasted chlorophyll at 1-month lead and S-NPP VIIRS chlorophyll for the period 2012–2015. White indicates that the correlation was not significant (*p* > 0.05).

To assess the uncertainties in our forecast, we utilize two deterministic skill metrics: ACC and RMSE. The ACC for the forecast was significant for the 1-month lead time (*R* = 0.33, *P* < 0.05) as well as for the 8- and 9-month lag forecast (*R* = 0.42 and *R* = 0.41 respectively, Table 2). This indicates that for these leads, the forecast chlorophyll had statistically the correct phasing when compared to those from S-NPP VIIRS. The spatial distribution of the anomaly correlation coefficient further reflects the overprediction of the upwelling in this Equatorial Pacific (Figure 6). While the forecasted chlorophyll concentrations at 1-month lead are significantly correlated with those from S-NPP VIIRS for the majority of the Equatorial Pacific, some areas in the upwelling tongue are not significant. The second skill metric, RMSE, increased from 0.040 μg chl L^−1^ at 1-month lead to 0.047 μg chl L^−1^ at 9-month lead forecast. These results suggest that while the phasing may have been reasonable at 8- and 9-month lag forecast, the amplitude of the signal was getting worse. Regardless, RMSE of 0.047 μg chl L^−1^ is still very acceptable for a 9-month lag forecast. These results suggest some skill in forecasting the chlorophyll variability in the Equatorial Pacific especially at 1-month lag when the ACC is significant and the RMSE is at its lowest. For all forecasts, the chlorophyll concentrations were always within 30.7% of the chlorophyll concentration from S-NPP VIIRS. This is similar to the uncertainties reported for this instrument (semi-interquartile range of S-NPP VIIRS versus in situ chlorophyll = 27.9%).

### Prediction of the 2015 El Niño

**FIGURE 7:**
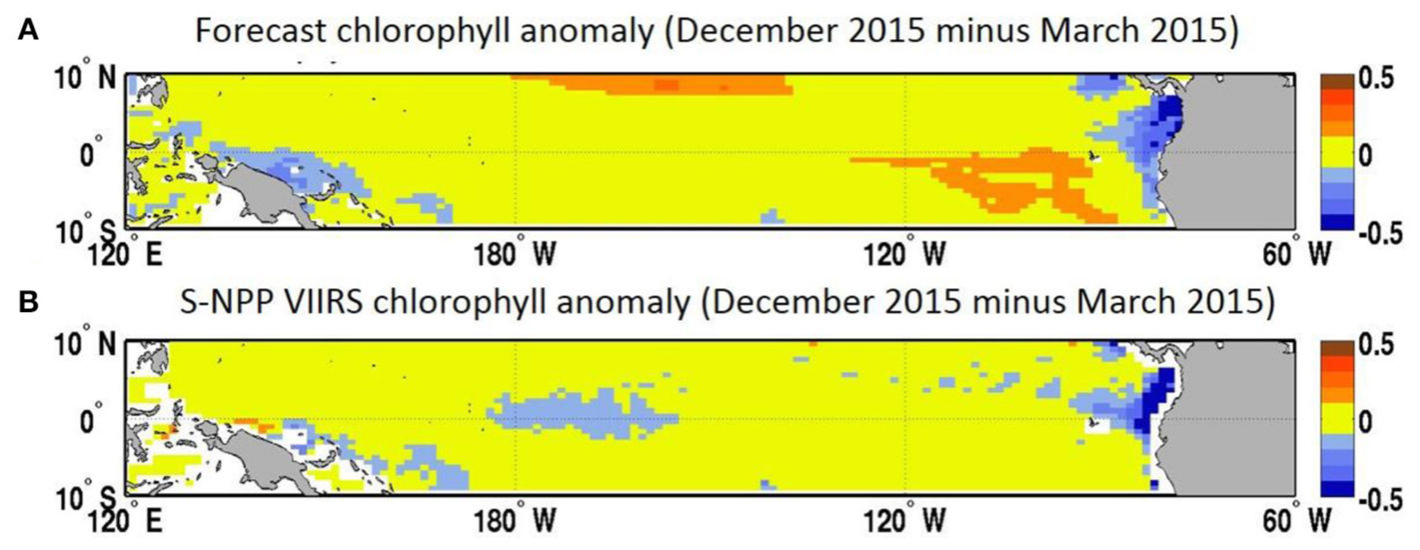
**(A)** Chlorophyll concentration anomaly (December 2015 minus March 2015, μg chl L^−1^) from the March 2015 forecast for December 2015 and **(B)** chlorophyll concentration from S-NPP VIIRS (μg chl L^−1^).

In the Equatorial Pacific, the El Niño Southern Oscillation is the dominant source of interannual variability and has been shown to have a considerable impact of the biogeochemistry, including chlorophyll concentration and recruitment of higher trophic levels, in this region (e.g., Strutton and Chavez, 2000; Martinez et al., 2009). Forecasting El Niño events is the focus of many prediction centers. While the focus of assessments such as the North American Multi-Model Ensemble home has been on the skills in forecasting sea surface temperature, there has been very little work on forecasting biogeochemical variables such as chlorophyll using a dynamical system. The temporal evolution of the various forecasts in this study highlights the variability between the forecasts and our skills in predicting the decline in chlorophyll concentration that was observed in the Equatorial Pacific during the 2015 El Niño event (Figure 4). Starting in January 2015 the forecast suggested a decline in chlorophyll concentration that would reach a minimum in May 2015 (average of the 8 forecasts available for this month of 0.13 μg chl L^−1^). The S-NPP VIIRS data observed this minimum 1 month later in June 2015 (0.13 μg chl L^−1^). The chlorophyll concentration from S-NPP VIIRS then increased to reach a peak in August 2015 (0.14 μg chl L^−1^). This increase in chlorophyll was also reflected in the various forecasts although it was overestimated. After August 2015, chlorophyll concentration declined reflecting the onset of the 2015 El Niño and the suppression of the upwelling in the Equatorial Pacific. This decline was also observed in the chlorophyll concentration from S-NPP VIIRS. Of the four forecasts available for September 2015, only one had predicted this decline. The other three forecasts predicted a decline but delayed by 1 month (chlorophyll started to decline in October 2015). For the four forecasts, September 2015 was their 6- to 9-month lead forecast which we previously showed had relatively low skills compared to the 1-month lead forecasts. In the last forecast (highlighted in red in Figure 3), September 2015 corresponded to its 6-month lead forecast and this forecast predicted particularly well the decline in chlorophyll concentration that occurred between August and December 2015 in the Equatorial Pacific in response to the El Niño event. The spatial distribution of the chlorophyll anomaly between December 2015 and March 2015 (first month of the last forecast available) coincides well with that from S-NPP VIIRS for the corresponding month (Figure 7). The area of negative anomaly in chlorophyll concentration along the South American coast is distinguishable in both the forecast and the S-NPP VIIRS chlorophyll data. The overestimation of the upwelling system in the forecast is also visible on this spatial representation of the chlorophyll anomalies. The temporal evolution of these various forecasts highlights the impacts that the atmospheric forcing data have on the forecast of chlorophyll. As the forecasts get closer to the El Niño event, the forecasted atmospheric and oceanographic physical forcing data have more skills and therefore lead to a better forecast in chlorophyll concentration. The forecast of chlorophyll in this region therefore relies heavily on the existence of accurate forecast of atmospheric forcing data. The initial conditions seem to play a more minor role in the forecasting skill for predicting chlorophyll in this region.

### Uncertainties of the Approach

The uncertainties in the forecast of atmospheric and oceanic variables used to force the model play a critical role in our ability to provide a successful forecast. The skill of the variables produced by the GMAO forecasting system and that are used to force the model in forecast mode can also be a source of uncertainties and have been assessed in (Borovikov et al., in review). The SST anomaly correlation coefficient from the forecast in the tropical Pacific has a high correlation coefficient (*R* > 0.8) with the Reynolds SST for lead month 1–3 and remained above 0.6 by lag month 9 indicating significant (*p* < 0.05) skill. A case study of the El Niño event of 2015/2016 in (Borovikov et al., in review) suggested an overprediction of the magnitude in SST anomalies observed during the 2015/2016 El Niño event but was overall in good agreement with the conditions that were observed.

The forecast of chlorophyll concentration presented here is based on one single set of forecasting data while the forecasting system used at GMAO provides forecasts for several ensembles. Using ensemble forecasting instead of a single forecast might further improve our skill. Initial conditions can be perturbed in various ways to account for initial condition uncertainty. The uncertainty in the forecasted forcing data provided by GMAO could be accounted for by running with the various ensembles they provide for the variables used to force the biogeochemical forecast. Finally the model uncertainty could be accounted for using some stochastic parametrization at the sub-grid level such as the one used by the European Centre for Medium Range Weather Forecasts (Buizza et al., 1999).

Another source of uncertainty in our forecast is the assimilation methodology, the Conditional Relaxation Analysis Method used for bias correction for SST products (Reynolds, 1988) and applied here for chlorophyll (Gregg, 2008). This method does not utilize ensembles which can potentially improve the initial conditions for the forecast. It would also extend the memory of the assimilation, which appears to survive <2 months here and assist in the skill of the 1-month forecast. However, there is little evidence that the 2–9 month forecasts could benefit substantially from improved initial conditions, which are quite close to the S-NPP VIIRS chlorophyll as suggested in Table 1.

### Future Improvements and Applications

While these results suggest some skill in our ability to forecast chlorophyll concentration in the Equatorial Pacific, they also highlight potential weaknesses and avenues for improvements. The skill of the forecasting system relies as previously mentioned on the bias in the model’s representation of physical and biogeochemical processes in the oceans, and the uncertainties in the forcing and assimilation data used. To further improve the forecasting system, each of these sources of bias and uncertainties needs to be assessed individually for weaknesses and possibilities for improvements. The range of applications of such a forecasting system, once properly set, can be extended for other variables. Applications include but are not limited to the prediction of Harmful Algal Blooms, fisheries, hypoxia/anoxia events, oil spills or the dispersal of pollutants. Prediction of temperature, ocean currents and velocities have for example been used for monitoring fisheries success, transport and spread of fish larvae, as well as seasonal fish migration (Johnson et al., 2005; Hobday and Hartmann, 2006; Bonhommeau et al., 2009). While the use of physical variables such as temperature, salinity and currents have been successfully used as covariates to explain distribution and catch rates of various species (e.g., Herron et al., 1989; Cole, 1999; Zagaglia et al., 2004; Bigelow and Maunder, 2007; Kaplan et al., 2016), these relationships can be limited since the behavior and recruitment of fish relies on changes in their prey concentration and composition. Accurate forecasts of the resources on which fish populations rely could provide the potential for strategic rather than reactive marine resource management during El Niño events for example. In the Equatorial Pacific, forecast of the effects of ENSO events on the physical conditions have been the subject of several studies starting in the 1980s (Cane et al., 1986). In the last two decades we have witnessed the development of two major El Niño events that had considerable impacts on both land and ocean conditions. The 1997–98 El Niño was particularly devastating for the ocean resources and led to the collapse of several fisheries and dramatic socio-economical repercussions for countries such as Peru. Anchovies, as well as other fisheries collapsed during both the 1982–83 and 1997–98 El Niño events. Forecasts such as the one presented here could therefore provide a framework to improve our management of resources during these events. Furthermore, the forecasting system presented here may provide a basis to expand the forecast from total chlorophyll to specific species including Harmful Algal Blooms. This could provide support for the management of many areas that need to monitor closely any development of harmful species in their waters. In the regions prone to Harmful Algal Blooms, such a forecast could also be used to improve the strategies to detect and manage most efficiently these events to minimize the repercussion on the human population and the associated economy.

## Figures and Tables

**TABLE 1:** Summary table of bias and uncertainties of the various elements of the system used to forecast.

Type of bias/uncertainties	Bias	Uncertainties
Chlorophyll from satellite versus in situ data (Global)	11.8%	*R* = 0.86, *P* < 0.05
Chlorophyll from free-run model versus satellite chlorophyll (transient forcing data, Equatorial Pacific, 2012–2015)	27.87 ± 1.72%	*R* = 0.72, *p* < 0.05
Chlorophyll from free-run model versus satellite chlorophyll (climatological forcing data, Equatorial Pacific, 2012–2015)	85.67 ± 2.77%	*R* = 0.47, *p* < 0.05
Chlorophyll concentration from assimilating run versus satellite chlorophyll (Equatorial Pacific, 2012–2015)	12.34 ± 0.52%	*R* = 0.95, *P* < 0.05

**TABLE 2:** Anomaly Correlation Coefficient (ACC) and RMSE between the chlorophyll concentration in the Equatorial Pacific from the forecast at 1- to 9-month lead time and the corresponding monthly chlorophyll concentration from S-NPP VIIRS.

No. months lead time	ACC	RMSE
1	0.329*	0.0399
2	0.272	0.0397
3	0.318	0.0411
4	0.267	0.0427
5	0.121	0.0435
6	0.153	0.0450
7	0.263	0.0470
8	0.417*	0.0471
9	0.409*	0.0472

* indicates that the anomaly correlation coefficient was significant (p < 0.05).
